# Fibrinogen monitoring does not significantly reduce rates of bleeding complications during catheter-directed thrombolysis

**DOI:** 10.1186/s42155-026-00682-y

**Published:** 2026-04-16

**Authors:** Minh A. Nguyen, Claire S. Kaufman

**Affiliations:** 1 School of Medicine, Oregon Health and Science University, Portland, OR USA; 2 Dotter Department of Interventional Radiology, Oregon Health and Science University, Portland, OR USA

## Abstract

**Purpose:**

To evaluate the impact of fibrinogen monitoring on bleeding complications and rates of premature tPA cessation in patients undergoing catheter-directed thrombolysis (CDT). We hypothesize that routine fibrinogen monitoring does not lower bleeding complications but may result in more cases of incomplete thrombolysis due to premature tPA termination.

**Methods:**

This single-institution retrospective study examined patients who underwent CDT for arterial, venous, or pulmonary thrombosis from 2011 to 2023. Rates of significant hemorrhages and premature tPA cessation were compared in patients with and without routine fibrinogen monitoring. Premature tPA cessation was defined as termination of thrombolytic infusion prior to the planned treatment endpoint due to low fibrinogen levels or clinical bleeding concerns.

**Results:**

A total of 355 CDT procedures met inclusion criteria, including 225 cases without routine monitoring (control group) and 130 with routine fibrinogen monitoring. Bleeding rates were similar between groups (3.55% vs. 3.07%, *p* = 1.00). However, premature tPA cessation occurred significantly more frequently in the fibrinogen monitoring cohort (17.5% vs. 1.78%, *p* < 0.001). Among cases with premature tPA cessation, residual thrombus requiring adjunctive interventions was common.

**Conclusion:**

This study demonstrated that routine fibrinogen monitoring was not associated with significantly reduced bleeding complications, but is linked to higher rates of premature tPA cessation in stable patients, which may contribute to incomplete thrombolysis and increased need for adjunctive interventions.

**Graphical Abstract:**

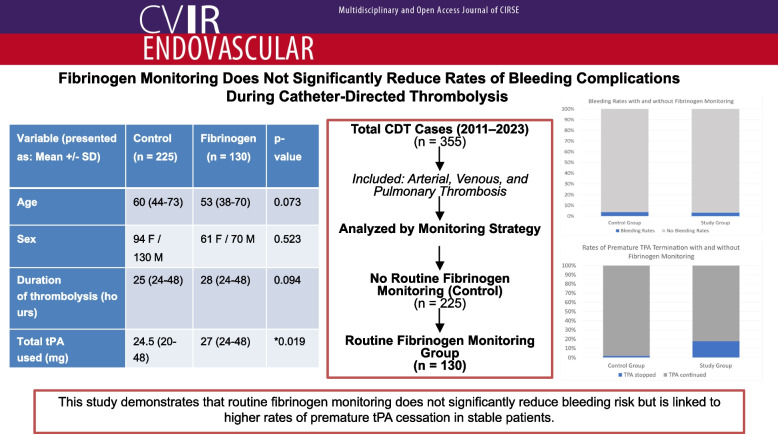

## Introduction

**Fig. 1:**
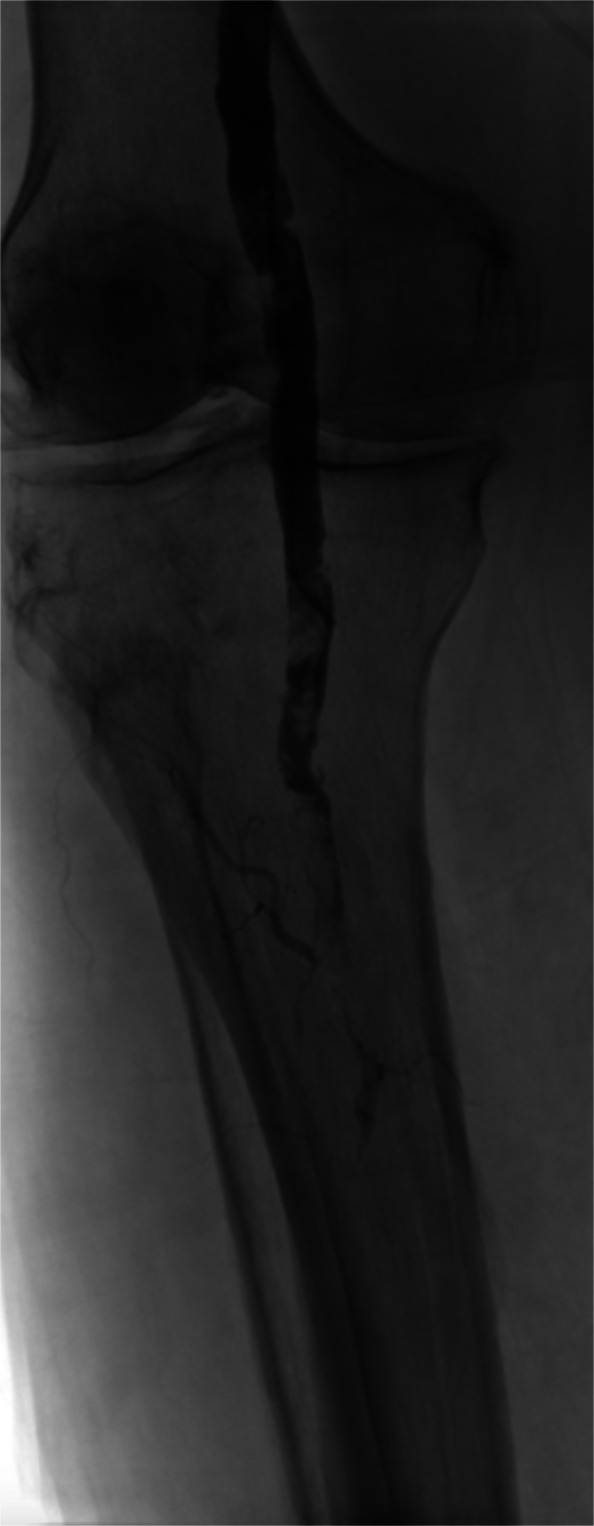
Angiographic image of the lower extremity showing a thrombus and occlusion of the popliteal artery

Catheter-directed thrombolysis (CDT) is a minimally invasive technique widely used for the treatment of arterial, venous, and pulmonary thrombotic disease [1, 2] (Fig. 1). Technical success rates for CDT vary from 80–85% to nearly 100%, depending on the definition used (thrombus clearance or placement of infusion catheter within the thrombus, respectively) [3, 4]. Although effective, CDT carries a risk of bleeding complications ranging from minor access site hematomas to life-threatening intracranial or gastrointestinal hemorrhage [5–8]. Reported rates of major bleeding vary depending on patient population, infusion protocols, and thrombolytic agents used.

**Fig. 2:**
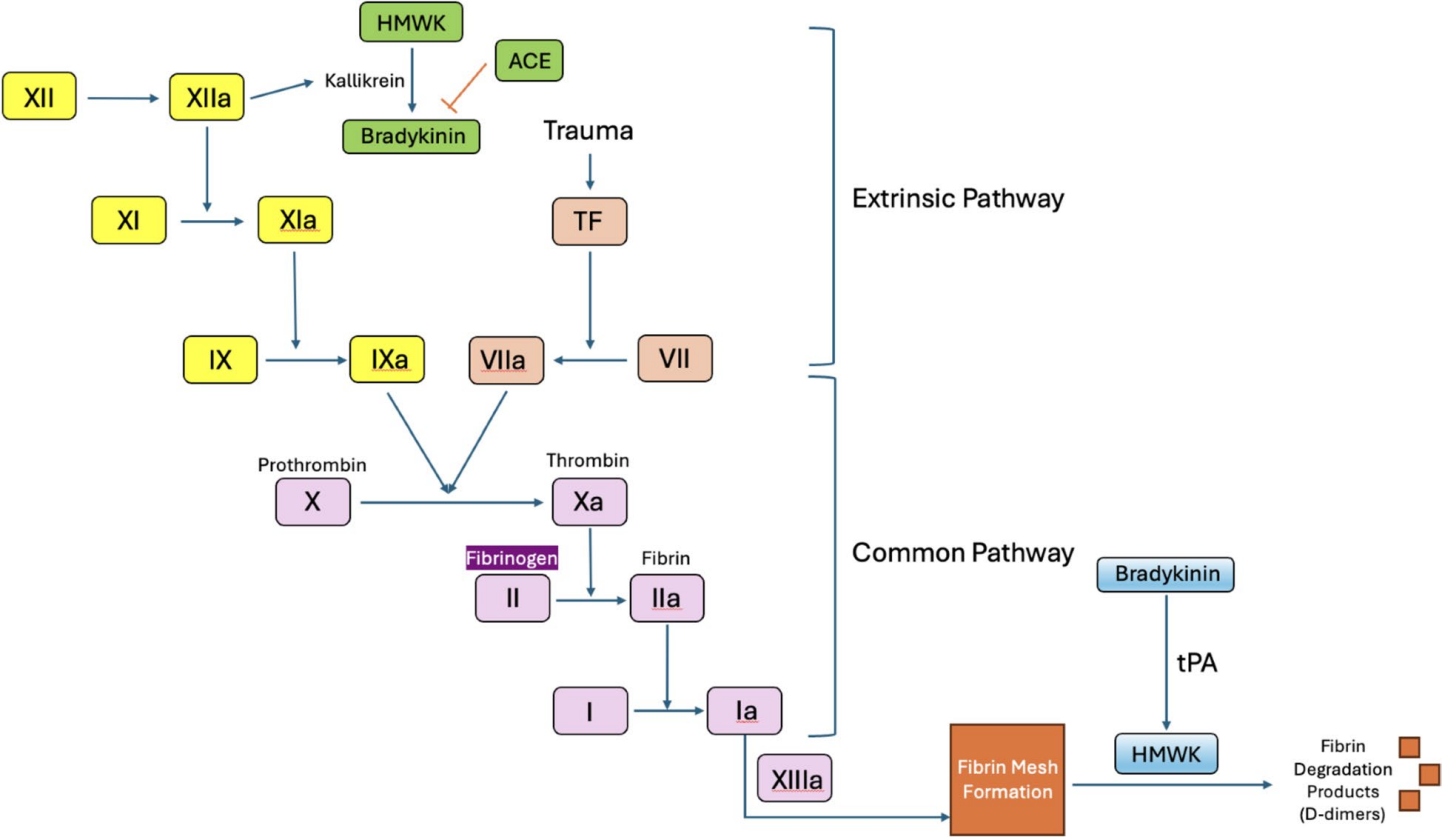
The coagulation cascade

Bleeding risks during CDT are multifactorial, with high dose of tissue plasminogen activator (tPA), prolonged infusion durations, and poorly controlled hypertension being notable risk factors [1, 9]. While other thrombolytic agents such as urokinase have historically been used, tPA was the primary thrombolytic agent used in the institutional protocols evaluated in this study. While CDT in venous thromboembolic disease (VTE) can reduce the severity of post-thrombotic syndrome, it carries an increased risk of bleeding compared to anticoagulation alone [3]. Efforts to mitigate these risks during CDT have historically focused on fibrinogen monitoring. Fibrinogen is a glycoprotein essential for thrombus stability and a key marker in the coagulation cascade (Fig. 2). During CDT, fibrinogen is degraded by tPA into fibrin degradation products, contributing to decreased systemic levels which can be easily monitored by lab draws [10, 11]. Because fibrinogen is the primary substrate for fibrin thrombus formation, markedly reduced plasma fibrinogen levels impair the ability to generate stable thrombi, which may predispose patients to increased bleeding risk.

While routine fibrinogen monitoring during CDT has been widely adopted, often with thresholds such as < 150 mg/dL prompting adjustments in thrombolytic dosing, its clinical utility and predictive value for major hemorrhagic risks remain controversial [12]. Emerging evidence suggests that fibrinogen degradation and significant reductions in its systemic levels occur in many CDT cases without a link to major hemorrhage or complications [7, 8]. There is inconsistent and uncompelling evidence linking low fibrinogen levels (< 150 mg/dL) to bleeding risks; however, absolute thresholds for stopping thrombolysis such as < 100 mg/dL or < 150 mg/dL remain widely used in clinical practice [11, 13]. Several studies have suggested that factors including high-dose tPA, forced infusion techniques, longer thrombolysis duration, and a relative (> 50%) reduction in fibrinogen may be more informative of bleeding risks than absolute thresholds [8, 14, 15]. This variability in clinical practice and outcomes underscores the need to critically evaluate the clinical utility of routine fibrinogen monitoring in CDT.

This study evaluated whether fibrinogen monitoring significantly reduced bleeding complications during CDT and investigated its impact on premature tPA cessation with subsequent procedural and patient outcomes. By analyzing data from a single-institution cohort over 13 years, this manuscript aims to provide clarity on the necessity and clinical implications of routine fibrinogen monitoring.

## Materials and methods

### Study design, setting, and population

This retrospective, single-institution study was performed in compliance with institutional IRB approval, evaluating all patients who underwent CDT for arterial, venous, or pulmonary thrombosis from January 2011 to January 2023. During this time, institutional practice gradually shifted from not monitoring fibrinogen levels during CDT to routine monitoring. While a precise date of transition cannot be delineated, the change was progressive and influenced in part by a sentinel patient event. This change allowed for the formation of two distinct cohorts: a control group (no fibrinogen monitoring) and a study group (routine fibrinogen monitoring). Inclusion criteria were patients who underwent CDT for arterial, venous, or pulmonary thrombosis between 2011 and 2023, treated with continuous intralesional tPA infusion. Exclusion criteria were procedures performed outside of the listed protocols (e.g., ischemic stroke) or incomplete clinical records. This study is reported in accordance with the STROBE guidelines for observational research.

### Procedure details

All CDT procedures were performed using standard techniques in the angiography suite, including the placement of an infusion catheter (EKOS or Cragg-McNamara) within or adjacent to the thrombus under fluoroscopic guidance. tPA was administered continuously via infusion; however, at times tPA was bolused intra-procedurally using pulse-spray delivery systems (boluses vary from 0 to 25 mg). Routine imaging follow-up, including computed tomography (CT) or venography, was performed to assess thrombus resolution if indicated. Patients undergoing CDT for pulmonary embolism often did not undergo follow-up imaging per institution protocol with catheters removed at bedside. Fibrinogen levels were monitored at the discretion of the treating physician and based on institutional protocol at the time of the procedure. The fibrinogen monitoring protocol uses standard laboratory tests (baseline tPA followed by q6hour unless tPA dose was adjusted or held then the lab draw interval decreased to q2hour).

### Data collection

Data were collected retrospectively from EMR and PACS databases. Variables obtained include tPA dose, infusion duration, premature cessation rates, fibrinogen levels, other hematologic labs, complications, cryoprecipitate administration, and clinical outcomes. Cryoprecipitate administration was recorded as the number of units transfused, with documentation of whether it was given in the context of tPA dose reduction or complete cessation. Cryoprecipitate was administered when fibrinogen levels fell below 100 mg/dL or in the presence of clinically evident bleeding, consistent with institutional practice during the study period. Clinical outcomes were defined as the need for additional interventions (e.g., thrombectomy, balloon maceration, or stenting), presence of residual thrombus at the time of lysis check, and ultimate procedural success or failure.

### Outcomes

The primary outcomes included rates of significant hemorrhage, defined as clinical presentations for intracranial hemorrhage or gastrointestinal bleeding, requiring transfusion or adjunctive interventions, or hemorrhages identified on imaging. Secondary outcomes included premature cessation of tPA infusion due to low fibrinogen or clinically significant hemorrhage, and subsequent need for additional interventions (e.g., residual thrombus). Residual thrombus was determined by the treating interventionalist at the time of lysis check based on fluoroscopic or CT venographic appearance, and further interventions were performed at the operator’s discretion. No uniform institutional threshold was applied.

### Definitions

Premature tPA cessation was defined as termination of thrombolytic infusion prior to the intended treatment endpoint due to low fibrinogen levels or clinical bleeding concerns. Residual thrombus was determined by the treating interventionalist at the time of lysis check.

### Statistical analysis

Categorical variables were analyzed using Pearson chi-square and Fisher’s exact tests. Statistical analyses were conducted using Excel and IBM SPSS. A *p* value < 0.05 was considered statistically significant.

## Results

**Fig. 3:**
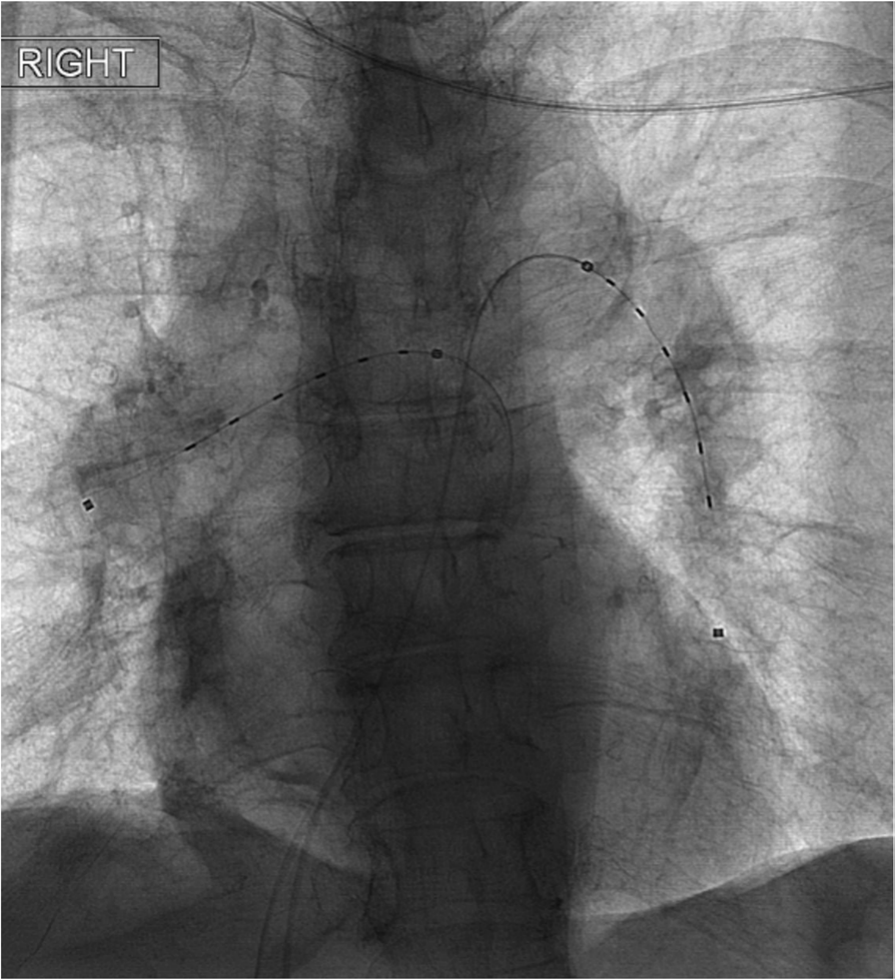
Fluoroscopic image demonstrating bilateral EKOS catheter placement within the pulmonary arteries for catheter-directed thrombolysis in the setting of acute PE

**Fig. 4:**
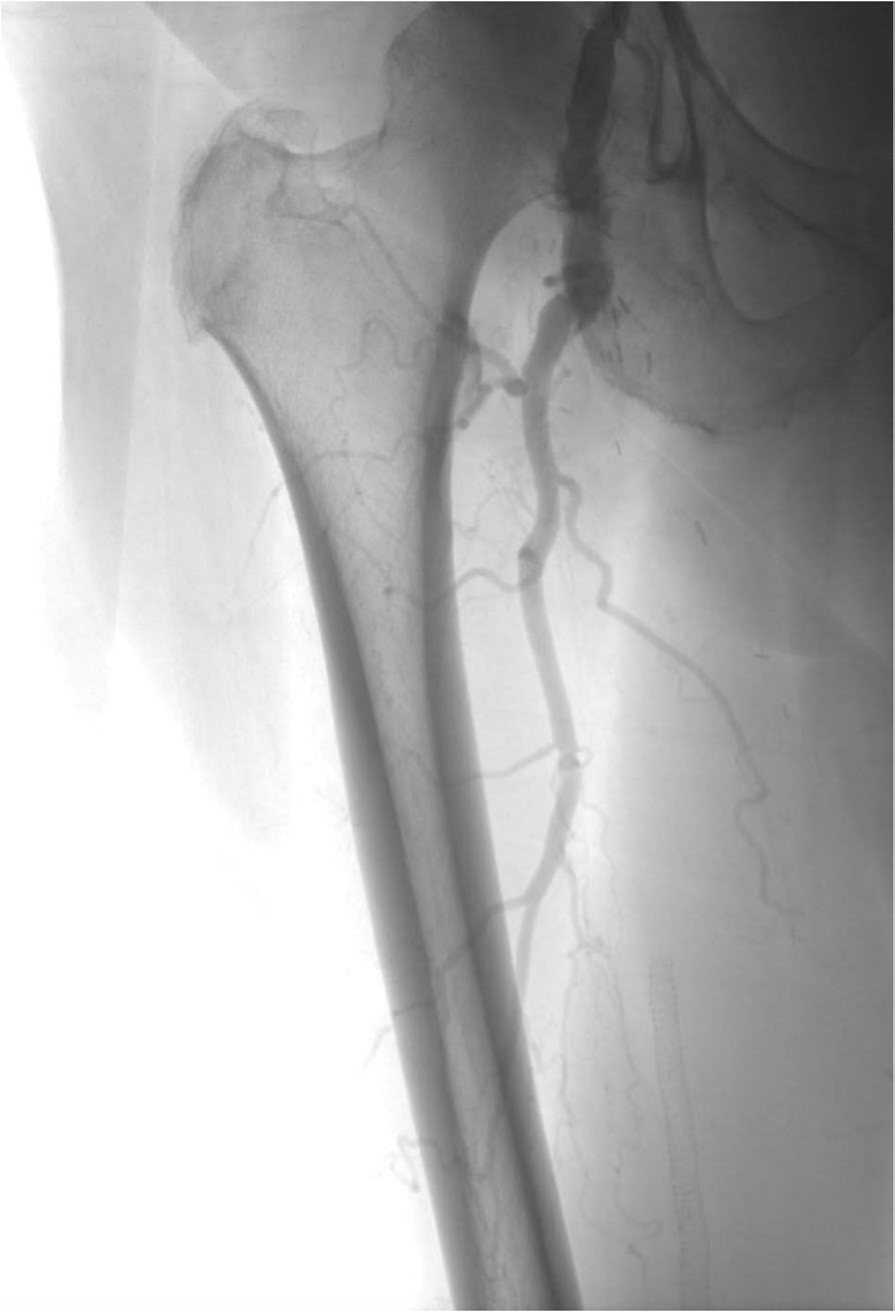
Oblique angiographic view of the left lower extremity showing abrupt cutoff of contrast opacification consistent with complete occlusion of the superficial femoral artery (SFA). Obtained prior to catheter-directed thrombolysis using a Cragg-McNamara infusion catheter

**Fig. 5:**
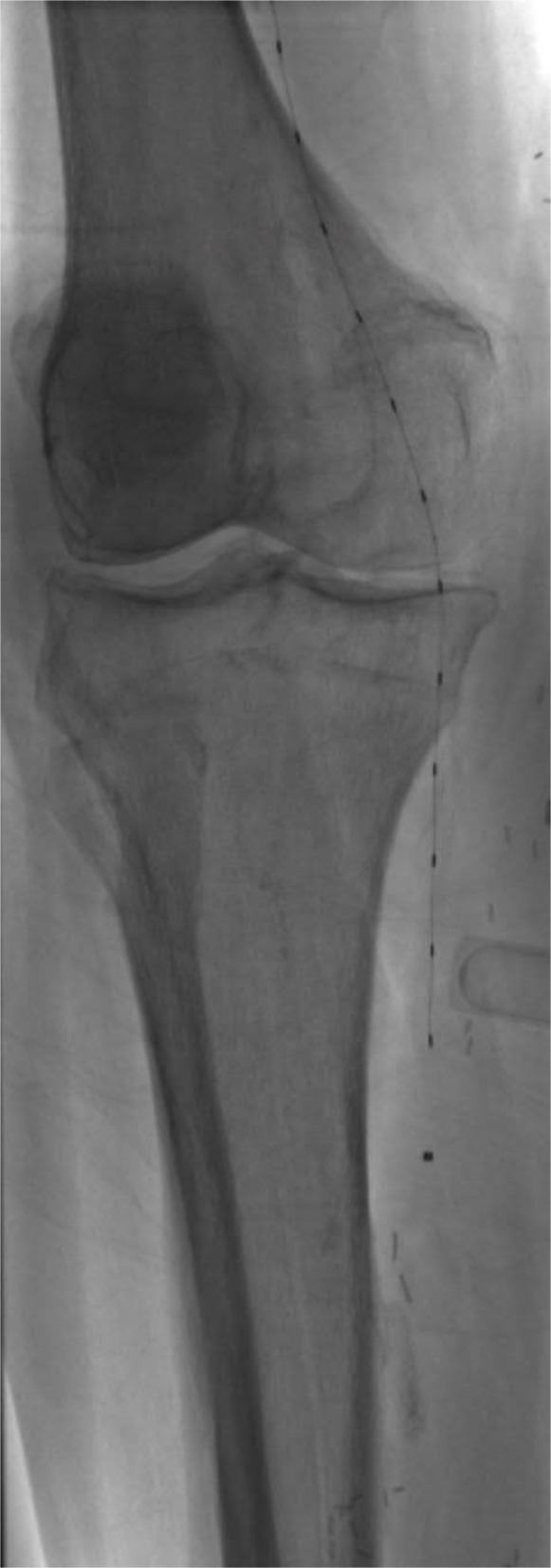
Anteroposterior fluoroscopic image demonstrating an EKOS catheter placement across the popliteal artery for catheter-directed thrombolysis. Radiopaque markers along the catheter outline the infusion zone, confirming appropriate positioning across the thrombus burden

A total of 355 CDT procedures met inclusion criteria. This includes 153 arterial, 167 venous, and 35 pulmonary artery thrombolysis cases (Figs. 3, 4 and 5) where patients received continuous tPA infusion for up to 72 h after catheter placement. Baseline demographic and clinical variables were compared between groups (Table 1). Of 355 cases, 225 cases constituted the control group where fibrinogen levels were not routinely monitored during thrombolysis. Our analysis defined this as cases without scheduled serial fibrinogen draws during thrombolysis after catheter placement. One hundred thirty cases were in the fibrinogen monitoring group, where fibrinogen levels were assessed at regular set intervals (every 6 h) during CDT to guide therapy, tPA dose, and duration of treatment.

**Table 1:** Baseline patient characteristics of control vs. fibrinogen groups

Variable	Control	Fibrinogen	*p* value
Age	60.0 [44.0–73.0] Range: 17.0–95.0	53.0 [38.0–70.0] Range: 0.0–100.0	0.073
Duration of thrombolysis (hours)	25.0 [24.0–48.0] Range: 4.0–72.0	28.0 [24.0–48.0] Range: 4.0–72.0	0.094
Total tPA used (mg)	24.5 [20.0–48.0] Range: 3.0–76.0	27.0 [24.0–48.0] Range: 2.0–81.0	0.019*
Sex	94 F/130 M	61 F/70 M	0.523
Type of thrombolysis	90 arterial/29 pulmonary/106 venous	63 arterial/6 pulmonary/61 venous	0.020*
Hypercoagulable state	179 N/46 Y	105 N/26 Y	1.000
History of malignancy	168 N/57 Y	107 N/24 Y	0.150
History of prior thrombotic events	136 N/89 Y	63 N/68 Y	0.027*
History of prior thrombolysis	190 N/35 Y	109 N/22 Y	0.766
Stents/IVC filter in location of thrombolysis	184 N/38 Y	93 N/36 Y	0.086

Continuous variables are presented as mean ± SD if normally distributed, or median [IQR] if non-normally distributed. Categorical variables are presented as counts. *p* values are calculated using Student’s *t*-test or Mann–Whitney *U* test for continuous variables, and chi-square or Fisher’s exact test for categorical variables

^*^A *p* value < 0.05 was considered statistically significant. Most variables show no significant differences (*p* > 0.05). Exceptions include Total tPA used (higher in fibrinogen group), Type of thrombolysis distribution, and History of prior thrombotic events

### Baseline characteristics

Baseline patient characteristics are summarized in Table 1. The control and fibrinogen monitoring groups were generally similar in terms of age, sex distribution, and prevalence of hypercoagulable states or malignancy. Differences were observed in several variables, including procedure type, history of prior thrombotic events, and total tPA dose, which were statistically higher in the fibrinogen monitoring cohort. These imbalances are important to acknowledge and are further discussed in the Discussion section.

### Bleeding rates

**Fig. 6:**
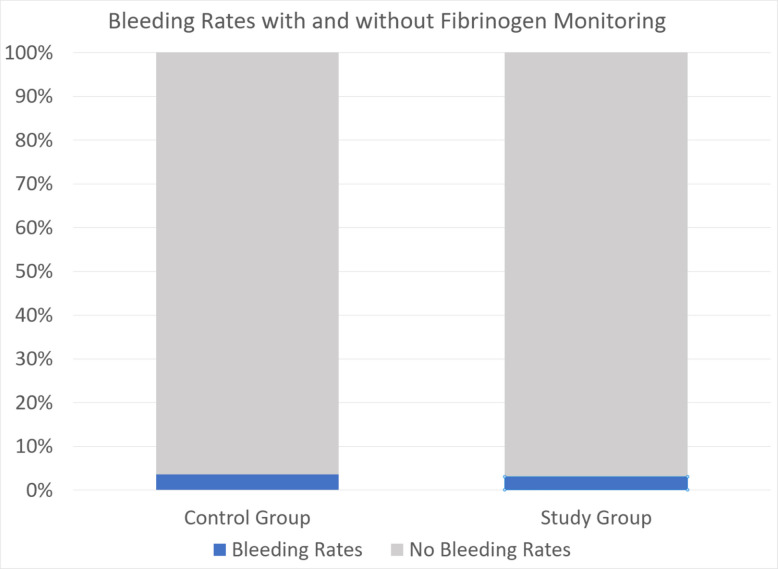
Bleeding rates (blue) in the control group vs. study group. 3.6% (8 cases) had significant bleeding in the control group, compared to 3.1% (4 cases) in the study group. No significant differences were found using statistical analysis, *p* = 1.00 using Fisher’s exact test (2-sided)

The overall rates of significant hemorrhage were low and showed no statistically significant difference between the two groups. In the control group, 3.55% (8 cases) experienced bleeding complications, compared to 3.07% (4 cases) in the fibrinogen monitoring group (*p* = 1.00) (Fig. 6). The types of bleeding complications vary from intracranial hemorrhage with midline shift (*n* = 1) to hypotension and drop in hematocrit necessitating a transfusion. Transfusion-threshold anemia (*n* = 8) was the most common type of complication (Table 2).

**Table 2:** Details of bleeding complications sorted by control vs. study groups, including procedure type, relevant PMH, units of pRBC transfused, and other complications. *CAD* coronary artery disease, *PAD* peripheral arterial disease, *AKI* acute kidney injury, *IVC* inferior vena cava, *MAP* mean arterial pressure

Group	Procedure type	Relevant medical history	Type of bleeding complication	Units of pRBC transfused	Other complications
Control (no monitoring), *n* = 8	Venous	CAD, stents/IVC filter in location of thrombolysis	Hypotensive, low MAP	2	None
	Venous	Malignancy, left common iliac vein compression	Epigastric pain and transfusion-threshold anemia	2	Fever
	Venous	Malignancy	Transfusion-threshold anemia	1	None
	Arterial	Antiphospholipid Syndrome	Transfusion-threshold anemia	1	Pain and AKI
	Arterial	Malignancy, PAD	Transfusion-threshold anemia	1	Continuous oozing from catheter site
	Venous	Atresia of infrarenal IVC	Hematemesis, transfusion-threshold anemia	1	Epistaxis
	Arterial	None	Intraparenchymal hemorrhage with midline shift	None	None
	Venous	Mesenteric ischemia	Hematochezia, transfusion-threshold anemia	2	None
Study (fibrinogen monitoring), *n* = 4	Arterial	Malignancy, CAD, PAD	Hypotensive, transfusion-threshold anemia	1	Acute neurological changes, rapid return to baseline
	Arterial	Atrial fibrillation, CAD, PAD	Hypotensive, groin hematoma	1	None
	Venous	Prior thrombotic events	Hemorrhagic shock of unknown source	3	Oozing from catheter site
	Arterial	Atrial fibrillation, PAD, carotid endarterectomy	Groin pseudoaneurysm, transfusion-threshold anemia, hypotension	None	None

### Premature tPA cessation

**Fig. 7:**
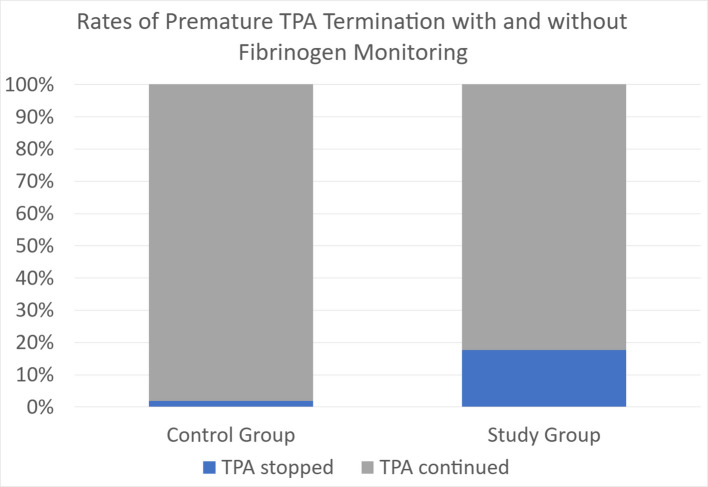
Rates of premature tPA cessation (blue) in the control group vs. study group. 1.78% (4 cases) had premature tPA cessation in the control group, compared to 17.5% (23 cases) in the study group. There was a statistically significant difference found using Pearson chi-square analysis, *p* < 0.001

Premature cessation of tPA occurred significantly more frequently in the fibrinogen monitoring group, at 17.5% (23 cases), compared to 1.78% (4 cases) in the control group (*p* < 0.001). This difference (Fig. 7) highlighted a potential unintended consequence of routine fibrinogen monitoring. There is an inherent difference in tPA cessation triggers among the two groups (laboratory fibrinogen values in the study group versus clinical factors in the control group), which can limit the direct interpretation of this statistical difference. This is elaborated further in the Discussion section.

### Outcomes of premature tPA cessation

**Fig. 8:**
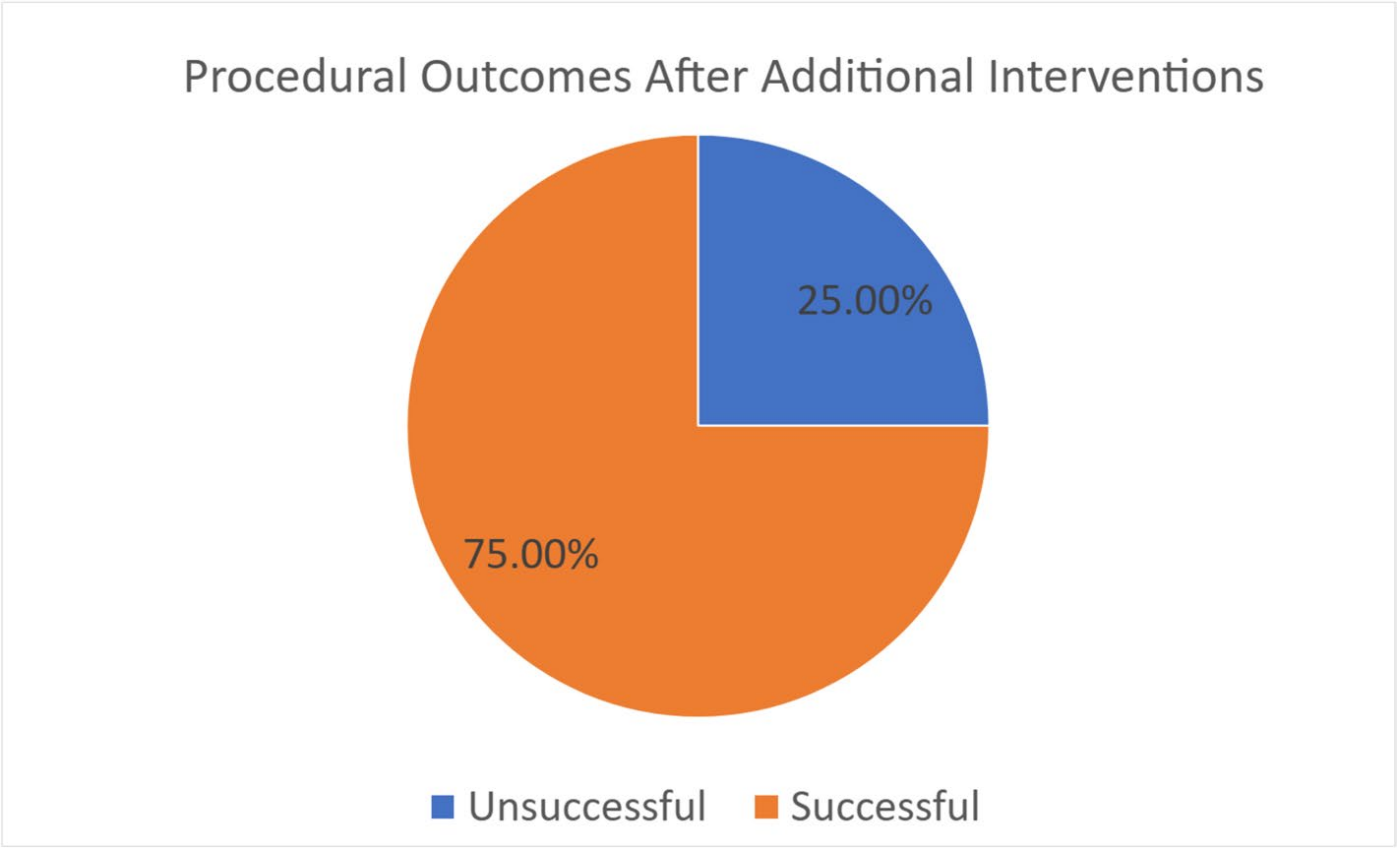
Procedural outcomes following additional interventions: 75% achieved eventual thrombus resolution or partial patency. Twenty-five percent were unable to complete lysis due to fibrinogen or had minimal improvement following additional interventions

Among the 27 total cases of premature tPA cessation across both groups (4 cases from the control group and 23 cases from the study group). Residual thrombi were observed in 20 cases (74%) as judged by the treating operator, leading to additional interventions including thrombectomy, balloon maceration, or stenting (depending on the underlying pathology). In the absence of a standardized definition across arterial, venous, and pulmonary cases, these determinations reflect real-world procedural decision-making. Six cases (25.9%) achieved complete thrombus resolution despite early termination of thrombolytic infusion. Five of 20 cases with residual thrombus (25%) resulted in complications (Fig. 8). In 3 of these 5 cases, thrombolytic infusion procedures were terminated prematurely due to low fibrinogen—with or without additional interventions such as thrombectomy, and patients were managed with systemic anticoagulation (Table 3). Two cases demonstrated persistent thrombus after 72 continuous hours of tPA infusion, and procedures were therefore terminated.

**Table 3:** Details on complications related to tPA cessation (*n* = 5)

Group	Additional interventions	Complications	Details
Control (no monitoring), *n* = 0	No applicable cases		
Study (fibrinogen monitoring), *n* = 5	None	Yes	Rethrombosis and additional occlusion were found on lysis check following tPA cessation and 4 units of cryoprecipitate. No further interventions pursued due to low fibrinogen
	None	Yes	Residual thrombus found on lysis check following tPA cessation and 1 unit of cryoprecipitate. Elected to terminate due to low fibrinogen
	Thrombectomy, angioplasty, and stenting	Yes	Due to low fibrinogen, tPA was halved following the first lysis check. There was an incomplete resolution of clot after 72 h due to premature tPA decrease. The patient ultimately rethrombosed and underwent subsequent procedures the following week despite resolution of therapeutic anticoagulation
	Thrombectomy	Yes	Due to low fibrinogen, tPA was halved following the first lysis check and 1 unit of cryoprecipitate given. There was incomplete resolution of clot and minimal improvement observed after 72 h due to premature tPA decrease
	Thrombectomy	Yes	Residual thrombus found on lysis check after tPA cessation, 2 units of cryoprecipitate, and thrombectomy. Elected to terminate due to low fibrinogen

### Cryoprecipitate administration

**Fig. 9:**
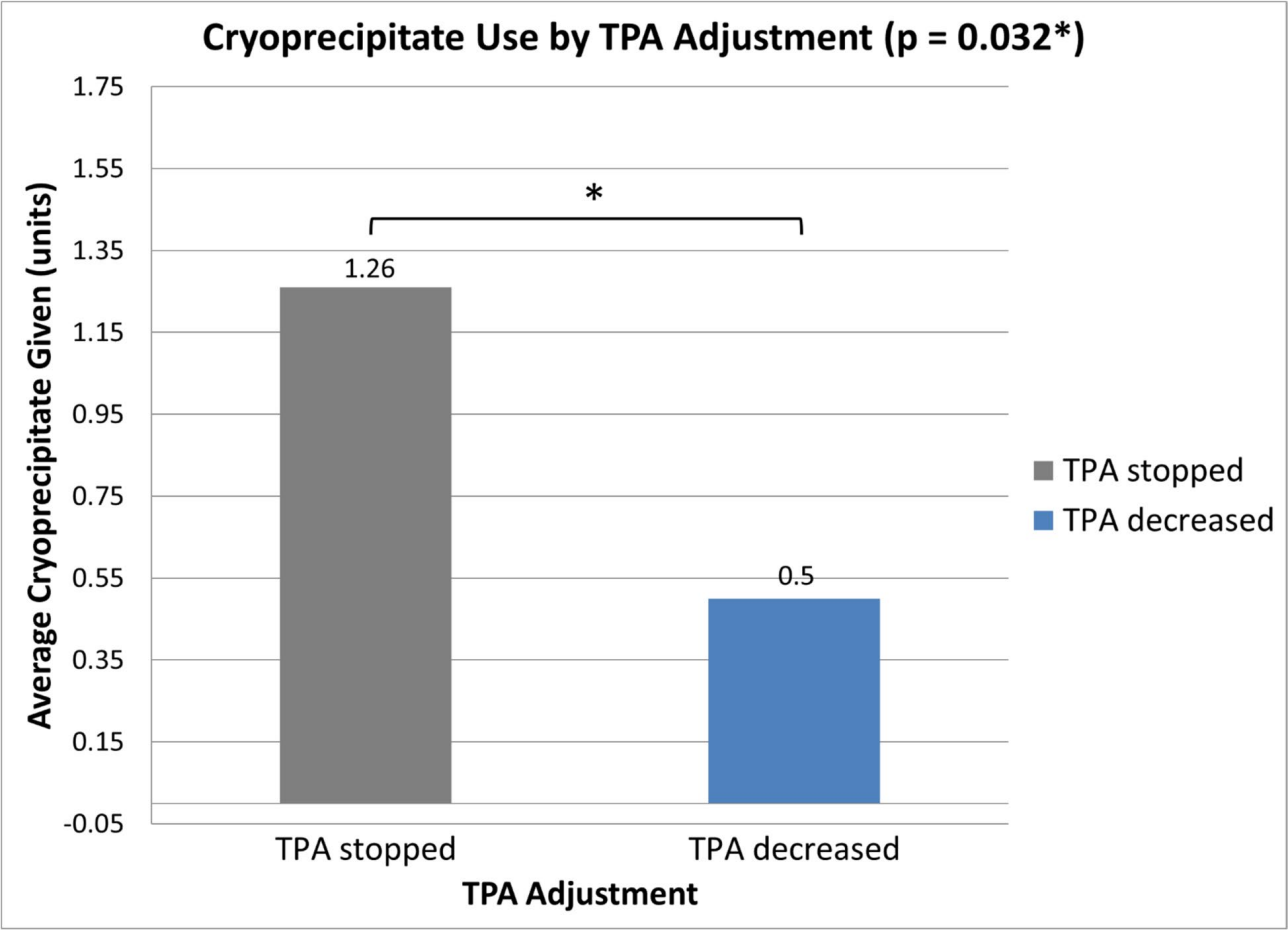
Mean cryoprecipitate dose given by tPA decrease vs. tPA cessation status. Patients whose tPA was stopped received an average of 1.26 units of cryoprecipitate, while those with decreased tPA dose received 0.50 units. This difference was statistically significant (*p* = 0.032)

**Fig. 10:**
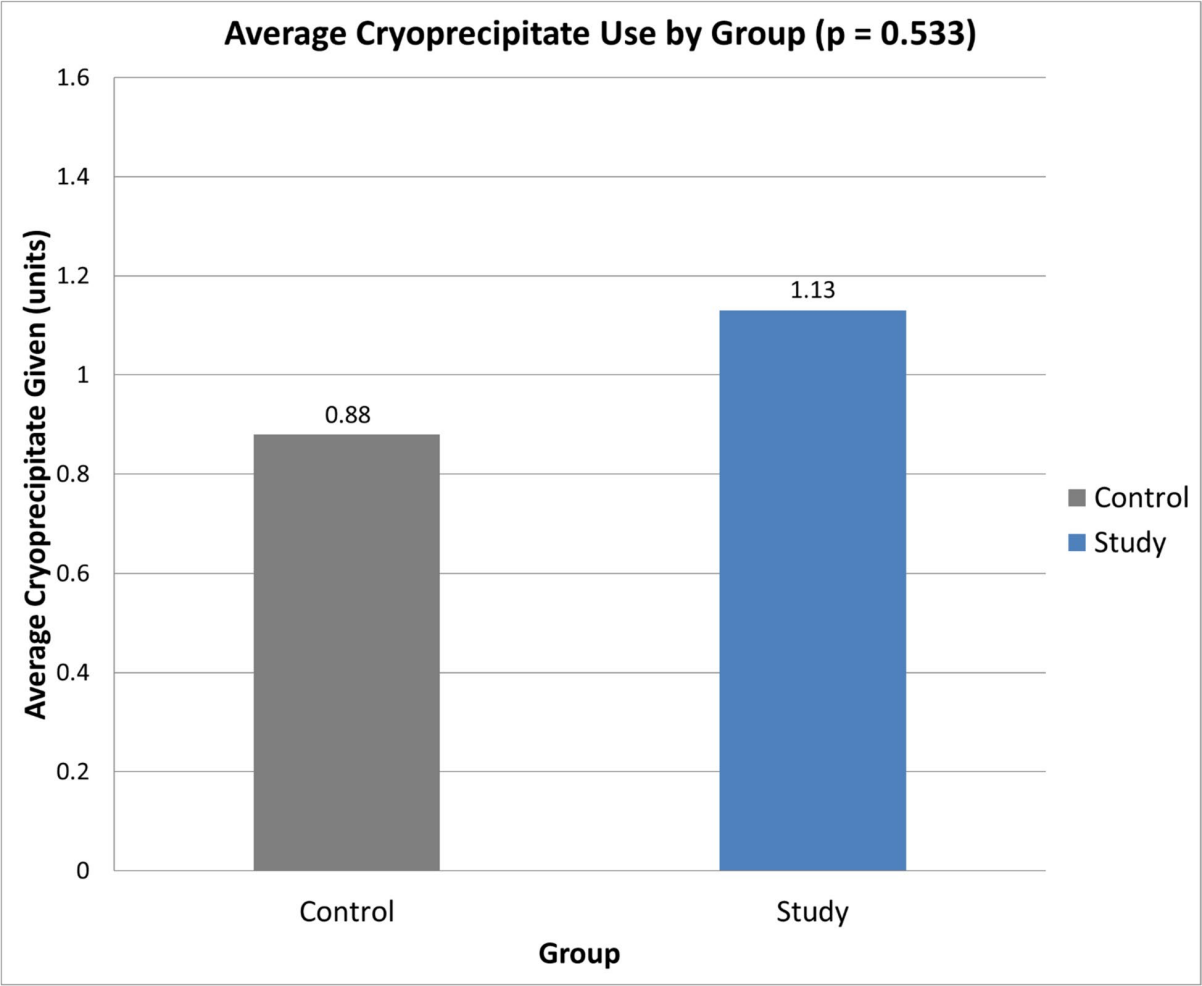
Mean cryoprecipitate dose administered in the control vs. study group. The control group received an average of 0.88 units of cryoprecipitate, while the study group received 1.13 units. There was no statistically significant difference (*p* = 0.533)

Cryoprecipitate was administered based on institutional protocol, which includes either low fibrinogen thresholds or clinical bleeding. In cases where tPA was prematurely halted or decreased, cryoprecipitate was administered in 19 out of 27 cases (70.3%) (Table 4). tPA was decreased, rather than stopped, in 6 out of 27 cases (22.2%). Of these 6 cases, cryoprecipitate was administered in 3 cases (50%) for a mean of 0.5 units per case. tPA was stopped in 21 out of 27 cases (77.8%). Of these 21 cases, cryoprecipitate was administered in 16 cases (76.2%) for a mean of 1.26 units per case. A quantitative comparison further demonstrated that patients whose tPA was stopped received significantly more cryoprecipitate (1.26 units) than those whose tPA dose was decreased (0.50 units; *p* = 0.032) (Fig. 9). While patients in the study group received a higher mean cryoprecipitate dose (1.13 units) than those in the control group (0.88 units), this difference was not statistically significant (*p* = 0.533) (Fig. 10).

**Table 4:** Details on outcomes of premature tPA decrease/cessation and amount of cryoprecipitate given by group

Group	tPA cessation details	Reason for tPA cessation	Cryoprecipitate given (and amount if given)	Residual thrombus requiring further interventions*	Additional interventions/note	Complications despite additional interventions
Control (no monitoring), *n* = 4	Stopped	Bleeding**** and fibrinogen level (64)	Not given	Yes	Thrombectomy and stenting	None
	Stopped	Fibrinogen level (36)*****	Yes (1 U)	Yes	Suction thrombectomy, angioplasty, and stenting	None
	Stopped	Bleeding	Yes (1 U)	No	None	None
	Stopped	Bleeding	Yes (unsure quantity)	**Patient developed significant ICH prior to lysis check		
Study (fibrinogen monitoring), *n* = 23	Stopped	Fibrinogen level (35)	Yes (4 U)	Yes	None	Yes***
	Stopped	Fibrinogen level (75)	Not given	Yes	Suction thrombectomy, balloon maceration, and stenting	None
	Stopped	Fibrinogen level (77)	Yes (1 U)	No	None	None
	Stopped	Fibrinogen level (35)	Not given	Yes	AngioJet and balloon maceration	None
	Stopped → restarted	Fibrinogen level (35)	Not given	No	None	None
	Stopped	Fibrinogen level (35)	Not given	No	None	None
	Stopped	Fibrinogen level (65)	Yes (1 U)	Yes	Balloon maceration	None
	Stopped	Fibrinogen level (84)	Yes (1 U)	Yes	Thrombectomy and stenting	None
	Stopped	Fibrinogen level (57)	Yes (1 U)	Yes	None	Yes***
	Stopped	Fibrinogen level (86)	Yes (1 U)	Yes	Stenting	None
	Stopped	Fibrinogen level (79)	Yes (3 U)	Yes	Aspiration thrombectomy and stenting	None
	Stopped	Fibrinogen level (35)	Yes (2 U)	Yes	Mechanical thrombectomy, balloon angioplasty	None
	Stopped	Fibrinogen level (66)	Yes (3 U)	Yes	Mechanical thrombectomy, balloon angioplasty, and stenting	None
	Stopped	Fibrinogen level (75)	Yes (2 U)	Yes	Femoral endarterectomy	None
	Stopped	Fibrinogen level (80)	Yes (1 U)	Yes	Rheolytic thrombectomy, angioplasty, and stenting	None
	Decreased	Fibrinogen level (74)	Yes (1 U)	Yes	Stenting	None
	Decreased	Fibrinogen level (145)	Not given	Yes	Thrombectomy, angioplasty, and stenting	Yes***
	Decreased	Fibrinogen level (83)	Yes (1 U)	Yes	Angioplasty and stenting	None
	Decreased	Fibrinogen level (98)	Not given	Yes	Thrombectomy, angioplasty, stenting	None
	Decreased	Fibrinogen level (104)	Not given	No	None	None
	Decreased	Fibrinogen level (93)	Yes (1 U)	Yes	Thrombectomy	Yes***
	Stopped	Fibrinogen level (53)	Yes (2 U)	Yes	Thrombectomy	Yes***
	Stopped	Fibrinogen level (93)	Yes (1 U)	No	None	None

^*^Further interventions include procedures such as thrombectomy, or balloon maceration performed routinely during lysis check

^**^Defined as > 50% luminal narrowing or thrombosis on lysis check

^***^Details on cases with complications related to tPA cessation (*n* = 5) provided in Table 3

^****^This was a postpartum patient who developed vaginal bleeding likely related to anticoagulation. Due to clinical concern, TPA was briefly stopped, and fibrinogen was subsequently checked at the 18-h mark and was found to be abnormal

^*****^Fibrinogen level was ordered at the first lysis check (24 h post-procedure) by the supervising attending and found to be low. There was no clinically evident bleeding or complications. However, as there were no fibrinogen labs ordered within the 6 h post-procedure, this case does not meet the criteria to be included in our fibrinogen monitoring group

## Discussion

Fibrinogen monitoring during catheter-directed thrombolysis (CDT) has been debated, with its utility in predicting and reducing bleeding complications uncertain. This study added to the evidence that fibrinogen monitoring does not significantly impact bleeding outcomes during CDT yet is associated with more premature tPA cessation, which can result in incomplete thrombolysis. This aligns with prior studies showing no strong correlation between fibrinogen levels and bleeding risk, even at thresholds below 150 mg/dL [7, 8]. Although low fibrinogen has been linked to systemic fibrinolysis during thrombolytic therapy, absolute levels often fail to capture the dynamic nature of hemostasis [10, 11]. Individual variability in fibrinogen degradation, compensatory mechanisms, and comorbid inflammation may explain these inconsistencies [10].

Despite this uncertainty, fibrinogen monitoring remains widespread, reported in approximately 82% of practitioners, despite mounting evidence of limited clinical utility [12]. Absolute thresholds such as < 150 mg/dL are still used to guide dosing, but supporting data are inconsistent [7, 8]. Recent studies suggest relative fibrinogen level reduction, rather than absolute value, may better predict bleeding risk [14], yet this approach adds complexity to treatment algorithms and may inadvertently lead to incomplete thrombolysis.

More concerning is the increased rate of premature tPA decrease or cessation with monitoring. Nearly 18% of monitored patients had tPA prematurely halted, and 6 had it decreased, compared with 2% in controls. Most required additional interventions for residual thrombus, which introduce further risks and resource burden, including higher costs, longer hospitalizations, and procedural complications [12]. Cryoprecipitate was administered more frequently and in greater amounts when tPA was stopped than when reduced, further underscoring the resource implications of monitoring-driven interruptions. In this study, cryoprecipitate administration likely reflects laboratory-triggered intervention in response to low fibrinogen levels, rather than definitive evidence that transfusion was clinically required. Routine fibrinogen monitoring also introduces some practical considerations, including repeated laboratory testing, increased venipuncture during thrombolytic infusion, and limited overnight availability of fibrinogen assays at some institutions, which may delay clinical decision-making.

Major hemorrhagic complications in CDT remain a key concern, with rates of 8.8 to 16% depending on protocol and patient factors [5, 6]. High tPA doses, prolonged infusions, and uncontrolled hypertension are known risk factors [1, 9]. In this study, bleeding rates were similar between monitored and unmonitored groups, supporting prior reports suggesting that fibrinogen levels alone may not reliably predict hemorrhagic complications during thrombolysis [7, 13]. Early tPA cessation may have theoretically prevented bleeding events that might have occurred if thrombolysis had continued in some monitored cases. Because this retrospective analysis cannot determine counterfactual outcomes, these findings should be interpreted cautiously. Baseline characteristics between cohorts were generally similar, although differences in prior thrombosis, procedure type, and total tPA dose likely reflect practice variability rather than systematic differences but remain potential confounders. While fibrinogen monitoring may prevent theoretical bleeding events, in our cohort it did not translate into lower complication rates and may have compromised thrombolytic efficacy.

Premature tPA cessation has important procedural implications. Of these patients, almost one-third (74%) had residual thrombi requiring further interventions. While adjunctive procedures represent standard endovascular management strategies rather than strictly negative procedural outcomes, they do reflect greater procedural complexity and resource utilization. Residual thrombus assessment and re-intervention decisions were operator-dependent without uniform thresholds, limiting comparability of outcomes.

Interpretation of the tPA cessation findings must also consider differences in treatment paradigms between cohorts. Cessation criteria differed between groups. In the monitoring cohort, thrombolysis was triggered by laboratory fibrinogen values, often without associated clinical or imaging evidence of bleeding. Whereas in the non-monitoring cohort, cessation was primarily based on clinical factors such as bleeding or hemodynamic instability. The difference in cessation rates therefore reflects two paradigms: laboratory-driven stoppage versus clinical instability. Although this distinction complicates interpretation, the finding remains robust—laboratory-triggered cessations frequently led to incomplete thrombolysis and a greater need for adjunctive interventions without a corresponding reduction in bleeding events. These findings suggest that routine fibrinogen monitoring, while well-intentioned, may alter treatment decisions in ways that influence thrombolysis completion without clearly improving patient safety.

## Limitations

This study has several limitations. As a retrospective single-institution analysis, the findings are subject to selection bias and may not be generalizable to other institutions with different thrombolysis protocols. Our institutional practice evolved from non-monitoring to routine fibrinogen monitoring during the study period, coinciding with other procedural and technological changes that may have influenced outcomes.

The study groups were not matched or propensity-adjusted, reflecting the retrospective nature of the analysis and the institutional transition in monitoring practice. Differences in baseline characteristics (Table 1) therefore remain potential confounders that may influence outcomes. The cohort included arterial, venous, and pulmonary thrombolysis cases. While these disease processes differ in pathophysiology and bleeding risk, fibrinogen monitoring protocols were applied uniformly across indications at our institution. Pooling these cases therefore reflects real-world clinical practice but introduces heterogeneity that may limit disease-specific interpretation.

Interpretation of the results is also influenced by differences in cessation thresholds between groups and the possibility that early cessation may have prevented theoretical bleeding events in some monitored patients. Residual thrombus burden was not consistently documented in cases where thrombolysis was completed as planned, limiting systematic comparison between completed and prematurely terminated thrombolysis cases. Interpretation of cryoprecipitate use results may also be limited by surveillance bias. Because cryoprecipitate was administered based on either low laboratory fibrinogen values or clinical evidence of bleeding, its use was more likely to be triggered in the monitoring group, where fibrinogen levels were measured systematically.

Prospective studies with standardized thrombolysis protocols are therefore needed to further validate and support these findings. Future work may identify more reliable hemorrhagic-risk biomarkers during CDT. Biomarkers such as D-dimer, platelet-function assays, or thromboelastography may provide more comprehensive assessments of coagulation dynamics [7, 8]. Randomized controlled trials comparing outcomes with and without fibrinogen monitoring (stratified by thrombolytic dosing, technique, and individual risk factors) are needed. The role of direct thrombolytics such as plasmin also warrants exploration [16].

## Conclusion

While fibrinogen monitoring did not reduce bleeding rates during CDT, it was associated with a higher rate of premature tPA cessation, increased need for adjunctive interventions, and greater resource utilization, including higher cryoprecipitate administration. While intended to improve safety, routine monitoring in our cohort failed to reduce bleeding and instead introduced practice changes that compromised thrombolytic efficacy.

## Data Availability

Available from the corresponding author upon reasonable request.
